# The detection of a strong episignature for Chung–Jansen syndrome, partially overlapping with Börjeson–Forssman–Lehmann and White–Kernohan syndromes

**DOI:** 10.1007/s00439-024-02679-w

**Published:** 2024-05-24

**Authors:** Niels Vos, Sadegheh Haghshenas, Liselot van der Laan, Perle K. M. Russel, Kathleen Rooney, Michael A. Levy, Raissa Relator, Jennifer Kerkhof, Haley McConkey, Saskia M. Maas, Lisenka E. L. M. Vissers, Bert B. A. de Vries, Rolph Pfundt, Mariet W. Elting, Johanna M. van Hagen, Nienke E. Verbeek, Marjolijn C. J. Jongmans, Phillis Lakeman, Lynne Rumping, Danielle G. M. Bosch, Antonio Vitobello, Christel Thauvin-Robinet, Laurence Faivre, Sophie Nambot, Aurore Garde, Marjolaine Willems, David Genevieve, Gaël Nicolas, Tiffany Busa, Annick Toutain, Marion Gérard, Varoona Bizaoui, Bertrand Isidor, Giuseppe Merla, Maria Accadia, Charles E. Schwartz, Katrin Ounap, Mariëtte J. V. Hoffer, Marjan M. Nezarati, Marie-José H. van den Boogaard, Matthew L. Tedder, Curtis Rogers, Alfredo Brusco, Giovanni B. Ferrero, Marta Spodenkiewicz, Richard Sidlow, Alessandro Mussa, Slavica Trajkova, Emma McCann, Henry J. Mroczkowski, Sandra Jansen, Laura Donker-Kaat, Floor A. M. Duijkers, Kyra E. Stuurman, Marcel M. A. M. Mannens, Mariëlle Alders, Peter Henneman, Susan M. White, Bekim Sadikovic, Mieke M. van Haelst

**Affiliations:** 1 Amsterdam UMC, Department of Human Genetics, University of Amsterdam, Meibergdreef 9, 1105 AZ Amsterdam, The Netherlands; 2 Amsterdam Reproduction & Development Research Institute, Amsterdam, The Netherlands; 3 Verspeeten Clinical Genome Centre, London Health Sciences Centre, London, ON N6A 5W9 Canada; 4 Department of Pathology and Laboratory Medicine, Western University, London, ON N6A 3K7 Canada; 5 Department of Human Genetics, Research Institute for Medical Innovation, Radboud University Medical Center, 6525 GA Nijmegen, The Netherlands; 6 Department of Genetics, University Medical Center Utrecht, 3584 CX Utrecht, The Netherlands; 7 Center for Medical Genetics, Antwerp University Hospital, University of Antwerp, Drie Eikenstraat 655, 2650 Edegem, Belgium; 8 Department of Clinical Genetics, Erasmus MC, University Medical Center Rotterdam, Rotterdam, The Netherlands; 9 Université de Bourgogne, Inserm U1231, Equipe GAD, Dijon, France; 10 CHU Dijon Bourgogne, FHU-TRANSLAD, Unité Fonctionnelle Innovation en Diagnostic Génomique Des Maladies Rares, 21000 Dijon, France; 11 CHU Dijon Bourgogne, Centre de Génétique, Centre de Référence Maladies Rares «Déficiences Intellectuelles de Causes Rares», FHU-TRANSLAD, Dijon, France; 12 CHU Dijon Bourgogne, Centre de Génétique, Centre de Référence Maladies Rares «Anomalies du Développement et Syndromes Malformatifs», FHU-TRANSLAD, Dijon, France; 13 INserm U1183, Department of Clinical Genetics, Montpellier University, 34090 CHU Montpellier, Montpellier, France; 14 Inserm U1245 and CHU Rouen, Department of Genetics and Reference Center for Developmental Disorders, Univ Rouen Normandie, 76000 Rouen, France; 15 Department of Medical Genetics, Timone Hospital, Marseille, France; 16 Genetics Department, University Hospital, UMR 1253, iBrain, University of Tours, Inserm, Tours, France; 17 APHP, Department of Genetics, Robert Debré Hospital, 75019 Paris, France; 18 Clinical Genetics and Neurodevelopmental Disorders, Centre Hospitalier de L’Estran, 50170 Pontorson, France; 19 Service de Génétique Médicale, CHU de Nantes, 44000 Nantes, France; 20 Laboratory of Regulatory and Functional Genomics, Fondazione IRCCS Casa Sollievo Della Sofferenza, San Giovanni Rotondo, Foggia, Italy; 21 Department of Molecular Medicine and Medical Biotechnology, University of Naples Federico II, Via S. Pansini 5, 80131 Naples, Italy; 22 Servizio di Genetica Medica, Ospedale Cardinale G. Panico, Tricase, LE Italy; 23 Department of Pediatrics and Human Development, College of Human Medicine, Michigan State University, Grand Rapids, MI 49503 USA; 24 Department of Clinical Genetics, Genetic and Personalized Medicine Clinic, Tartu University Hospital, Tartu, Estonia; 25 Department of Clinical Genetics, Institute of Clinical Medicine, University of Tartu, Tartu, Estonia; 26 Department of Clinical Genetics, Leiden University Medical Center, Leiden, The Netherlands; 27 Genetics Program, North York General Hospital, Toronto, ON M2K 1E1 Canada; 28 Greenwood Genetic Center, Greenwood, SC 29646 USA; 29 Department of Medical Sciences, University of Torino, Via Santena 19, 10126 Turin, Italy; 30 Unit of Medical Genetics, Città Della Salute e Della Scienza Hospital, Turin, Italy; 31 Department of Clinical and Biological Science, University of Torino, Turin, Italy; 32 Service de Génétique, CRMR AnDDI-Rares, CHU Reims, Reims, France; 33 Department of Medical Genetics and Metabolism, Valley Children’s Hospital, Madera, CA USA; 34 Department of Public Health and Pediatric Sciences, University of Torino, Turin, Italy; 35 Pediatric Clinical Genetics Unit, Regina Margherita Childrens’ Hospital, Turin, Italy; 36 Liverpool Center for Genomic Medicine, Liverpool Women’s Hospital, Liverpool, UK; 37 Department of Pediatrics, Le Bonheur Children’s Hospital, Memphis, TN USA; 38 Division of Genetics, Department of Pediatrics, University of Tennessee Health Science Center, Memphis, TN USA; 39 Victorian Clinical Genetics Services, Murdoch Children’s Research Institute, Parkville, VIC 3052 Australia; 40 Department of Paediatrics, University of Melbourne, Melbourne, Australia; 41 Amsterdam UMC, Department of Paediatrics, Emma Children’s Hospital, University of Amsterdam, Meibergdreef 9, 1105 AZ Amsterdam, The Netherlands; 42 Amsterdam UMC, Emma Center for Personalized Medicine, Amsterdam, The Netherlands

## Abstract

Chung-Jansen syndrome is a neurodevelopmental disorder characterized by intellectual disability, behavioral problems, obesity and dysmorphic features. It is caused by pathogenic variants in the *PHIP* gene that encodes for the Pleckstrin homology domain-interacting protein, which is part of an epigenetic modifier protein complex. Therefore, we hypothesized that *PHIP* haploinsufficiency may impact genome-wide DNA methylation (DNAm). We assessed the DNAm profiles of affected individuals with pathogenic and likely pathogenic *PHIP* variants with Infinium Methylation EPIC arrays and report a specific and sensitive DNAm episignature biomarker for Chung–Jansen syndrome. In addition, we observed similarities between the methylation profile of Chung–Jansen syndrome and that of functionally related and clinically partially overlapping genetic disorders, White–Kernohan syndrome (caused by variants in *DDB1* gene) and Börjeson–Forssman–Lehmann syndrome (caused by variants in *PHF6* gene). Based on these observations we also proceeded to develop a common episignature biomarker for these disorders. These newly defined episignatures can be used as part of a multiclass episignature classifier for screening of affected individuals with rare disorders and interpretation of genetic variants of unknown clinical significance, and provide further insights into the common molecular pathophysiology of the clinically-related Chung–Jansen, Börjeson–Forssman–Lehmann and White–Kernohan syndromes.

**Supplementary Information:**

The online version contains supplementary material available at 10.1007/s00439-024-02679-w.

## Introduction

Chung-Jansen syndrome (CHUJANS; OMIM #617,991) is an autosomal dominant inherited neurodevelopmental disorder (NDD) caused by heterozygous pathogenic variants in *PHIP*, located on chromosome 6q14.1 (OMIM #612,870). CHUJANS is characterized by developmental delay (DD), intellectual disability (ID), behavioral problems, overweight/obesity and specific facial features, in combination with other symptoms, such as vision problems, hypotonia and gastrointestinal problems (Ligt et al. 2012; Webster et al. 2016; Jansen et al. 2018; Craddock et al. 2019).

*PHIP* encodes the Pleckstrin homology domain-interacting protein (PHIP) and derives its name from the binding and interaction with the Pleckstrin homology (PH) domain of Insulin Receptor Substrate 1 and 2 (IRS-1 and IRS-2) proteins. PHIP enhances insulin mediated processes (Farhang-Fallah et al. 2002, 2000), but also proopiomelanocortin (POMC) transcription (Marenne et al. 2020). POMC promotes anorexigenic signals and is a key component of the leptin-melanocortin pathway, which is vital for maintaining energy homeostasis (Coll et al. 2004). Disturbance of this leptin-melanocortin pathway explains the susceptibility to develop obesity in individuals with CHUJANS.

**Fig. 1:**
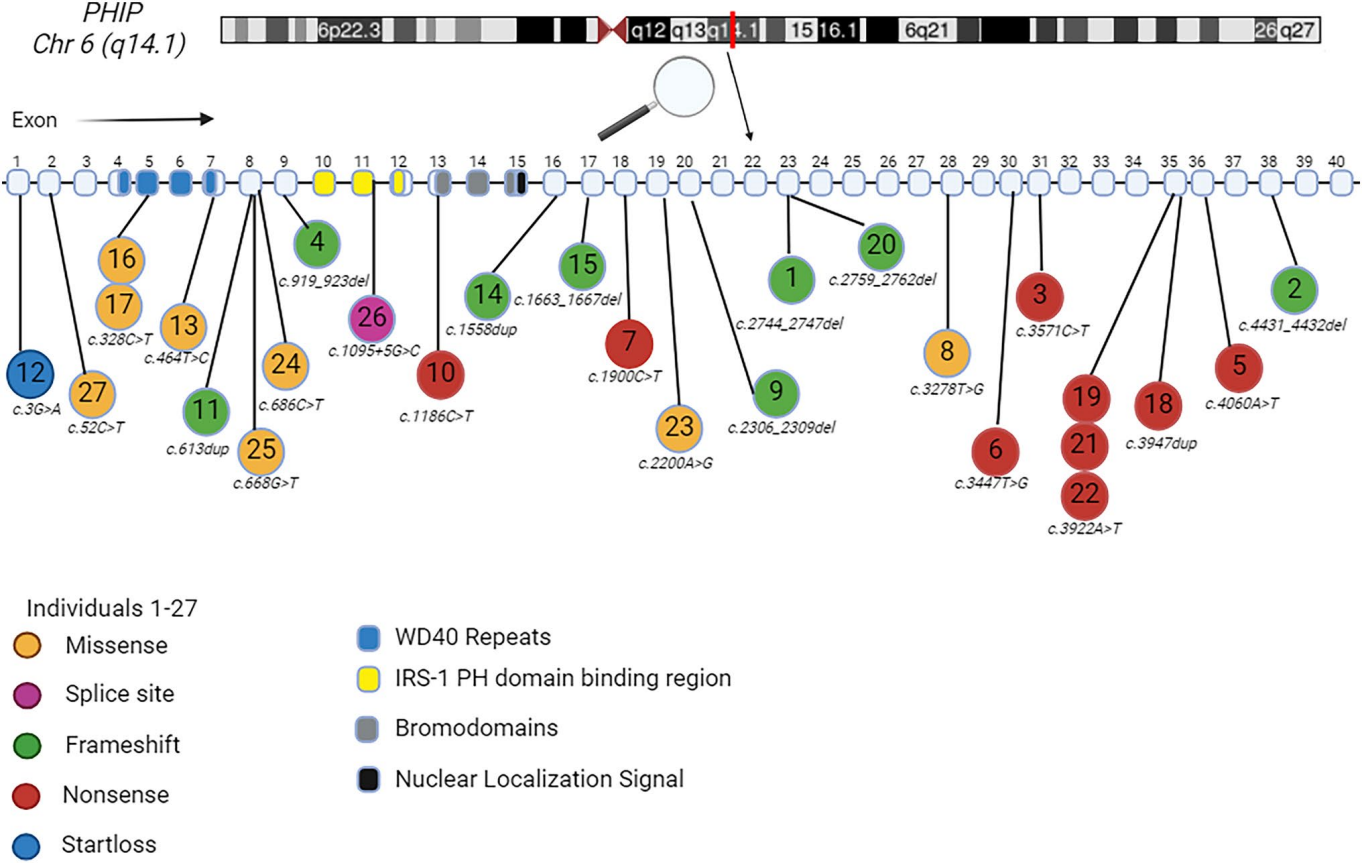
Schematic overview of *PHIP*, showing exons 1–40 and the different PHIP regions/domains. WD40 repeats are encoded by exons 4–7 (blue), PH domain binding regions by exons 10–12 (yellow), bromodomains by exons 13–15 (grey) and NLS by exon 15, like described in Jansen et al*.* 2018 and Craddock et al*.* 2019 (Jansen et al. 2018; Craddock et al. 2024). This overview shows the primary samples (affected individuals 1–13) and validation samples (affected individuals 14–27) and the different types of *PHIP* variants; missense (orange), splice site (purple), frameshift (green), nonsense (red) and start loss (blue) variants. Created with BioRender.com

Multiple PHIP isoforms have been distinguished (Farhang-Fallah et al. 2002, 2000; Podcheko et al. 2007). The largest PHIP isoform, also known as PHIP1, is localized in the cell nucleus and is widely expressed in brain and various body tissues. It comprises eight WD40-repeats, two bromodomains, a PH domain-binding region and presumably one or two nuclear localizing signals (NLS) (Craddock et al. xxxx; Podcheko et al. 2007; Jin et al. 2006)[Fig. 1]. The eight WD (Tryptophan(W) – Aspartic Acid(D)) 40-repeats act as scaffolds that facilitate protein–protein interactions. WD40-repeat containing proteins are known to be involved in a wide variety of cellular and molecular mechanisms (Podcheko et al. 2007; Neer et al. 1994; Schapira et al. 2017; Smith et al. 1999). Bromodomains recognize and bind acetylated lysine on histone and non-histone proteins (‘epigenetic reading’) and are involved in various processes (histone modification, chromatin remodeling and transcriptional regulation) that have an effect on gene expression (Podcheko et al. 2007; Arrowsmith et al. 2012; Filippakopoulos et al. 2012). Moreover, PHIP has been identified as one of the DDB1-CUL4-associated factors (DCAFs) and is also known as DCAF14. As a DCAF, PHIP serves as a substrate receptor for Cullin-RING ligase complex 4 (CRL4). CRL4s are ubiquitin E3 ligase protein complexes that add ubiquitin to target proteins (ubiquitination) and so exert a wide array of functions (Jin et al. 2006; Townsend et al. 2021).

The latter described functional PHIP domains clearly indicate a role in epigenetic regulation machinery. Therefore, we hypothesized that deleterious variants in the *PHIP* gene may alter genome-wide DNA methylation (DNAm) (Laan et al. 2022; Bjornsson 2015). Genome-wide DNAm signatures (episignatures) have thus far been reported for over 70 genetic conditions (Levy et al. 2022a). DNAm episignatures have recently been adapted for clinical testing as sensitive and specific biomarker for screening of individuals with suspected rare disorders and for interpretation of genetic variants of unknown clinical significance (Sadikovic et al. 2021).

In this study, we aimed to (Ligt et al. 2012) define an episignature for CHUJANS, (Webster et al. 2016) validate this episignature in a cohort of affected individuals with rare *PHIP* variants (VUS to pathogenic variants) and (Jansen et al. 2018) compare the CHUJANS episignature with other known and previously reported episignature disorders.

## Materials and methods

### Ethics statement

The study has been approved by the Amsterdam University Medical Centers Medical Ethics Commission (W20_193) and the Western University Research Ethics Board (REB 106302 and 116,108).

### Informed consent statement

Verbal and written informed consent for encoded use of DNA and medical information was obtained from affected individuals and/or their families.

### Samples for the CHUJANS episignature

DNA samples were extracted from whole blood of 26 affected individuals with likely pathogenic or pathogenic *PHIP* variants and one affected individual with a *PHIP* variant of uncertain significance (VUS). Classifications were stated as reported by the involved genome diagnostic laboratories and based on American College of Medical Genetics (ACMG) guidelines (Richards et al. 2015). Of these 27 affected individuals carrying *PHIP* variants in total, nine had a nonsense, eight had a missense, eight had a frameshift, one had a splice site and one had a start loss variant. A slight majority of included affected individuals (15/27) were female (Fig. 1 and Table 1). The age of affected individuals ranged from 2 to 47 years old at time of blood drawing.

**Table 1:** Molecular features of CHUJANS study cohort

#	Variant (*PHIP*; NM_017934.7)	Variant type	Class	Sex
1	c.2744_2747del p.(Lys915Serfs*15)	FS	LP	F
2	c.4431_4432del p.(Phe1478Leufs*19)	FS	LP	M
3	c.3571C > T p.(Gln1191*)	NS	LP/P	M
4	c.919_923del p.(Ile307Profs*22)	FS	LP/P	F
5	c.4060A > T p.(Arg1354*)	NS	LP/P	F
6	c.3447 T > G p.(Tyr1149*)	NS	LP/P	F
7	c.1900C > T p.(Gln634*)	NS	LP/P	F
8	c.3278 T > G p.(Leu1093Arg)	Mis	LP	M
9	c.2306_2309del p.(Pro769Leufs*43)	FS	LP	F
10	c.1186C > T p.(Arg396*)	NS	P	F
11	c.613dup p.(Cys205Leufs*10)	FS	P	F
12	c.3G > A p.(?)	SL	LP	M
13	c.464 T > C p.(Leu155Pro)	Mis	LP	F
14	c.1558dup p.(Cys520Leufs*6)	FS	LP	M
15	c.1663_1667del p.(Gln555Valfs*4)	FS	P	M
16	c.328C > T p.(Arg110Cys)	Mis	LP	M
17	c.328C > T p.(Arg110Cys)	Mis	LP	F
18	c.3947dup p.(Tyr1316*)	NS	P	F
19	c.3922A > T p.(Arg1308*)	NS	P	M
20	c.2759_2762del p.(Arg920Asnfs*10)	FS	P	F
21	c.3922A > T p.(Arg1308*)	NS	P	F
22	c.3922A > T p.(Arg1308*)	NS	P	F
23	c.2200A > G p.(Ser734Gly)	Mis	VUS	M
24	c.686C > T p.(Ser229Leu)	Mis	LP	M
25	c.668G > T p.(Gly223Val)	Mis	LP	M
26	c.1095 + 5G > C p.?	Splice	LP	M
27	c.52C > T p.(Leu18Phe)	Mis	LP	F

^#^ = affected individual’s study number, *FS* Frameshift, *Mis* Missense *NS* Nonsense, *SL* Start loss, *Splice* Splice Site, *VUS* Variant of uncertain significance, *LP* likely pathogenic, *P* pathogenic, *M* Male, *F* Female

Samples from affected individuals 1–13 (annotated in light grey) were used as a training cohort for determination of the episignature. Samples from affected individuals 14–27 (annotated in dark gray) were used as a testing and a validation cohort. Variant classification was done according to the ACMG guidelines (Richards et al. 2015)

Samples were obtained through an international multicenter collaboration between institutions in The Netherlands, Canada, Belgium, France, Italy, United States of America, United Kingdom, Estonia and Australia.

### DNAm profiling and quality control

DNAm profiles were acquired using Illumina Infinium MethylationEPIC BeadChip microarrays (San Diego, CA) as previously described (Aref-Eshghi et al. 2021). The DNAm data quality control analysis procedure has been described in detail in previous studies (Aref-Eshghi et al. 2018a, 2019, 2021). In brief, the obtained methylated and unmethylated signal intensities were analyzed in R (V 4.1.2). Illumina normalization was applied with background correction using the minfi package (V 1.40.0) (Aryee et al. 2014). Arrays with > 5% probe failure rate were excluded from further analyses. Moreover, probes that met the following criteria were excluded from downstream analysis: those located on the allosomes, those containing SNPs near or at the CpG interrogation or single nucleotide extension locations, those recommended by Illumina to be eliminated, and those known to cross-react with chromosomal sites outside their target regions. To visualize the batch structure and to detect potential outliers, principal component analysis (PCA) was performed, but no outliers were detected.

### Selection of controls for CHUJANS episignature discovery

For episignature detection, the following protocol was used: Control samples were randomly selected from the EpiSign Knowledge Database (EKD, https://episign.lhsc.on.ca/knowledge_database.html) and matched to the affected individuals’ samples by age and sex using the MatchIt package (V 4.3.3) (Ho et al. 2007).

The beta values, ranging from zero (indicating no methylation) to one (indicating full methylation), representing methylation levels for each probe, were transformed into M-values using a logit transformation. These M-values were then utilized in a linear regression analysis using the limma package (V 3.50.0) to detect differentially methylated probes (DMPs) (Ritchie et al. 2015). In order to account for confounding variables, estimated blood cell proportions were included in the model matrix. The p-values were moderated using the eBayes function in the limma package (Houseman et al. 2012), and then corrected using the Benjamini and Hochberg (BH) algorithm.

A three-step process was used to select probes that most robustly distinguish affected individuals’ samples from controls. First, we selected $i=1000$ probes with the highest products of mean methylation differences between the affected individual and control groups and negative logarithms of corrected p-values, using the formula -|∆β| log *p* where ∆β equals the average of case beta values minus the average of control beta values. Second, using the caret package (V 6.0.91, Kuhn M. Caret package. J Stat Soft. 2008;28 (Farhang-Fallah et al. 2002):1–26), areas under the receiver operating curve (AUROC) were calculated and *j* = 500 probes with the highest AUROCs were retained. Finally, we eliminated probes with pair-wise Pearson’s correlation coefficients greater than k=0.75.

A total of 212 probes remained upon the latter procedure and underwent verification using hierarchical clustering, which was performed using Ward's method on Euclidean distance. Multidimensional scaling (MDS) was subsequently performed by scaling the pairwise Euclidean distances between samples. To assess the reproducibility of the episignature, 13 iterations of leave-one-out cross-validation (LOOCV) were performed. During each iteration, one of the affected individuals’ samples was used for testing, while the remaining affected individuals’ samples and matched control individuals were used for probe selection. MDS plots were generated for each iteration to observe whether the test sample clustered with the affected individuals’ samples.

### Construction of the binary classifier

Using the selected probes, we constructed a support vector machine (SVM) classification model. We constructed the model by training the affected individuals’ samples against matched control samples, 75% of other control samples from the EKD, and 75% of samples from other rare genetic disorders from the previously published EpiSign™ V3 clinical classifier within EKD. The remaining 25% of samples were used for testing the model. The model generates methylation variant pathogenicity (MVP) scores ranging from 0 to 1. Higher scores indicate greater similarity to the identified episignature.

It was noted that the identified CHUJANS episignature additionally had the capacity to distinguish samples of individuals with Börjeson-Forssman-Lehmann syndrome (BFLS; OMIM #301,900) and White-Kernohan syndrome (WHIKERS; OMIM #619,426) from controls. This observation pointed to a shared DNAm pattern between these disorders and CHUJANS. Consequently, we conducted further analyses to unveil a second episignature specific to the combined CHUJANS-BFLS-WHIKERS cohort, utilizing a total of 31 samples (13 CHUJANS, 14 BFLS, and 4 WHIKERS samples).

### Episignature discovery and model construction for the combined cohort of CHUJANS, BFLS, and WHIKERS

The aforementioned probe selection and model construction procedure were repeated for the combined CHUJANS-BFLS-WHIKERS cohort. Using 62 control samples matched to the 31 affected individuals’ samples by age and sex, we selected 213 probes as the defining episignature. The probe selection criteria are summarized in Table S1.

An SVM classifier was developed using the selected set of probes and the 31 samples from the combined cohort, following a methodology similar to that of the CHUJANS cohort. Additionally, 31 rounds of LOOCV were executed.

### Comparison with other episignature disorders

The degree of overlap between DMPs of CHUJANS, the combined cohort, and those of other rare genetic disorders with known episignatures from EKD was visualized, as previously described in detail (Levy et al. 2022b). For this section of the analysis, individuals were selected for the control group in a way that ensures that none of them have a syndrome with a known episignature. Moreover, only probes common between the 450 k and EPIC arrays were included in the analysis. DMPs were considered as probes with mean methylation differences > 5% between the affected individual and control groups and corrected p-values < 0.01.

We annotated DMPs using the annotatr package (V 1.20.0) along with AnnotationHub (V 3.2.1) and annotations hg19_cpgs, hg19_basicgenes, hg19_genes_intergenic, and hg19_genes_intronexonboundaries (Cavalcante and Sartor 2017). We applied agglomerative clustering to create a tree-and-leaf diagram, which visualized the degree of similarity in DNAm profiles between CHUJANS, the combined CHUJANS-BFLS-WHIKERS cohort, and other disorders with known episignatures previously reported as part of the EpiSign™ v3 classifier (Levy et al. 2022a). In the diagram, for each cohort, *n* DMPs with the lowest p-values were selected, where *n* is the minimum number of 500 DMPs in that cohort. We generated a matrix with probes as rows and cohorts as columns, with each entry showing the median of β-values at the corresponding probe for all samples within the cohort. We then performed Euclidean clustering using Ward's method on the resulting matrix. Finally, we plotted the tree-and-leaf diagram based on the Euclidean clustering using the TreeAndLeaf package (V 1.6.1). In order to detect differentially methylated regions (DMRs), we also conducted assessments using the R package DMRcate (V 2.12.0) (Peters et al. xxxx). The parameters were specified as having a minimum of 5 CpGs within a 1-kilobase proximity to each other, and a minimum absolute mean methylation difference of 0.05 between the affected individuals and controls. Moreover, we refined the outcomes by applying a Fisher P-value threshold of 0.01. Gene ontology (GO) enrichment analysis was performed on the identified DMRs using missMethyl package in R (V 1.30.0). However, no DMRs were found and GO analyses did not find enriched terms (data not shown).

## Results

### Episignature detection for CHUJANS

We evaluated the DNAm profiles (peripheral blood) of 13 affected individuals with clinical and molecular CHUJANS (Fig. 1 and Table 1: #1–13).

**Fig. 2:**
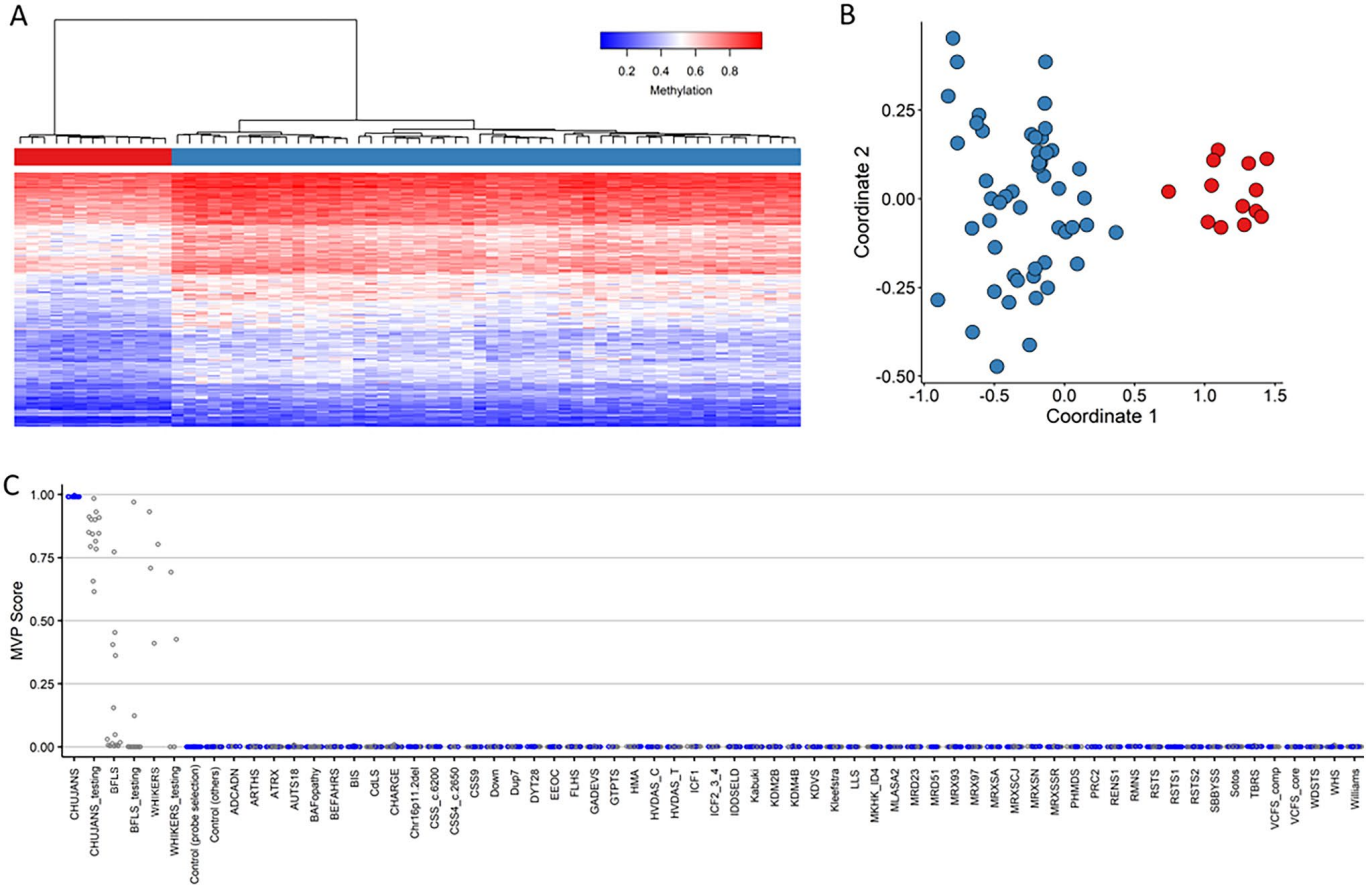
CHUJANS Episignature.** A** Hierarchical clustering was performed, where the rows represented the selected probes and columns represented the samples. The color scale illustrated the methylation levels from blue (0 or no methylation) to red (1 or full methylation). CHUJANS and control groups are labeled red and blue, respectively. Clear separation of the case and control groups was observed. **B** An MDS plot was generated, with red and blue circles representing the CHUJANS and control individuals, respectively. The plot depicted the distinct methylation pattern of CHUJANS from controls. **C** MVP scores were generated by the SVM classifier, with blue and grey circles representing training and testing samples, respectively. The low scores of testing samples from other disorders with known episignatures (with the exception of BFLS and WHIKERS samples) indicated the high sensitivity and specificity of the classifier. All CHUJANS validation samples received high MVP scores and were classified as CHUJANS. Using a cut-off value of 0.25 for the MVP score, all of the WHIKERS, two of the WHIKERS testing, four of the BFLS, and one of the BFLS testing samples were also positive for the CHUJANS episignature

Next, an independent cohort of 13 affected individuals with pathogenic or likely pathogenic *PHIP* variants, and one affected individual carrying a VUS in *PHIP,* were tested as a validation cohort (Fig. 1 and Table 1: #14–27). We used the 13 samples of individuals with CHUJANS (#1–13) and 52 matched control individuals for probe selection, and we assessed the robustness of the 212 identified probes (Table S2) by using hierarchical clustering and MDS (Fig. 2A and Fig. 2B). In order to assess the reproducibility of the CHUJANS episignature, we executed 13 rounds of LOOCV (Figure S1).

### Construction of the CHUJANS episignature classifier

To ensure more accurate classification of CHUJANS samples, we constructed an SVM using the 212 selected probes. We trained the SVM by using the 13 CHUJANS samples against the matched control samples, 75% of other controls, and 75% of samples from other disorders included in the EpiSign™ V3 classifier and preliminary cohorts from the EKD. We used the remaining 25% to test the classifier.

We observed that the identified CHUJANS episignature also matched several samples of affected individuals with BFLS (EpiSign™ V3) and WHIKERS (episignature not included in EpiSign ™ V3) (data not shown), indicating a significant overlap in methylation patterns among these syndromes. To account for this overlap and increase the model’s sensitivity to CHUJANS we utilized the BFLS and WHIKERS samples, as testing samples within the model (Fig. 2C, Table 1).

Furthermore, all study samples (i.e., the CHUJANS validation samples, along with the BFLS and WHIKERS samples), were integrated into the unsupervised models (hierarchical clustering and MDS) for testing purposes. The heatmap and MDS plots were generated with the 212 selected probes, and the additional study samples were plotted alongside the CHUJANS and controls used for the probe selection. The WHIKERS discovery samples, two of the WHIKERS testing samples, four of the BFLS discovery samples, and one of the BFLS testing samples showed variable degrees of similarity with the CHUJANS methylation pattern (Figure S2). Finally, to assess the reproducibility of the CHUJANS episignature, we executed 31 rounds of LOOCV (Figure S3).

### Construction of the classifier for the combined cohort of CHUJANS, BFLS and WHIKERS

Next, we used the CHUJANS, WHIKERS and BFLS discovery cohort samples all for probe selection to construct a combined episignature (Table 2).

**Table 2:** Molecular features of the BFLS (*PHF6*) and WHIKERS (*DDB1*) cohorts

#	Gene (Transcript)	Variant	Variant type	Class	Sex
28	*PHF6* (NM_001015877.2)	c.1024C > T, p.(Arg342*)	NS	P	M
29	*PHF6* (NM_001015877.2)	c.1022A > G, p.(Glu341Gly)	Mis	LP	M
30	*PHF6* (NM_001015877.2)	c.1021G > C, p.(Glu341Gln)	Mis	LP	M
31	*PHF6* (NM_001015877.2)	c.48dup, p.(Cys17Metfs*5)	FS	LP	M
32	*PHF6* (NM_001015877.2)	c.266G > A, p.(Gly89Glu)	Mis	LP	M
33	*PHF6* (NM_001015877.2)	c.769A > G, p.(Arg257Gly)	Mis	P	M
34	*PHF6* (NM_001015877.2)	c.1024C > T, p.(Arg342*)	NS	P	M
35	*PHF6* (NM_001015877.2)	c.134G > A, p.(Cys45Tyr)	Mis	P	M
36	*PHF6* (NM_001015877.2)	c.22A > T, p.(Lys8*)	NS	P	M
37	*PHF6* (NM_001015877.2)	c.2 T > C, p.?	SL	P	M
38	*PHF6* (NM_001015877.2)	c.134G > A, p.(Cys45Tyr)	Mis	P	M
39	*PHF6* (NM_001015877.2)	c.296G > T, p.(Cys99Phe)	Mis	LP	M
40	*PHF6* (NM_001015877.2)	c.1024C > T, p.(Arg342*)	NS	P	M
41	*PHF6* (NM_001015877.2)	c.1024C > T, p.(Arg342*)	NS	P	M
42	*PHF6* (NM_001015877.2)	c.860G > A, p.(Gly287Asp)	Mis	LP	F
43	*PHF6* (NM_001015877.2)	c.715C > T, p.(His239Tyr)	Mis	LP	F
44	*PHF6* (NM_001015877.2)	c.800C > T, p.(Thr267Ile)	Mis	LP	F
45	*PHF6* (NM_001015877.2)	c.1039C > T, p.(Arg347*)	NS	LP	M
46	*PHF6* (NM_001015877.2)	c.199A > G, p.(Ile67Val)	Mis	VUS	M
47	*PHF6* (NM_001015877.2)	c.827A > G, p.(Lys276Arg)	Mis	VUS	M
48	*PHF6* (NM_001015877.2)	c.434G > A, p.(Ser145Asn)	Mis	VUS	M
49	*PHF6* (NM_001015877.2)	c.890G > T, p.(Cys297Phe)	Mis	P	F
50	*PHF6* (NM_001015877.2)	c.266G > A, p.(Gly89Glu)	Mis	LP	M
51	*PHF6* (NM_001015877.2)	c.122C > T, p.(Ala41Val)	Mis	VUS	M
52	*DDB1* (NM_001923.5)	c.637G > A, p. (Glu213Lys)	Mis	P	M
53	*DDB1* (NM_001923.5)	c.637G > A, p. (Glu213Lys)	Mis	P	F
54	*DDB1* (NM_001923.5)	c.643G > A, p.(Glu215Lys)	Mis	P	F
55	*DDB1* (NM_001923.5)	c.562C > T, p.(Arg188Trp)	Mis	VUS	F
56	*DDB1* (NM_001923.5)	c.3169C > T, p.(Arg1057*)	NS	VUS	M
57	*DDB1* (NM_001923.5)	c.2683A > G, p.(Thr895Ala)	Mis	VUS	M
58	*DDB1* (NM_001923.5)	c.637G > A, p.(Glu213Lys)	Mis	P	M
59	*DDB1* (NM_001923.5)	c.562C > T, p.(Arg188Trp)	Mis	VUS	F

^#^ = affected individual study number, *FS* Frameshift, *Mis* Missense *NS* Nonsense, *SL* Start loss

*VUS* Variant of uncertain significance, *LP* likely pathogenic, *P* pathogenic, *M* Male, *F* Female

Samples from affected individuals 28–41 (light grey) were used as BFLS discovery samples. Samples from affected individuals 42–51 (dark grey) were used as BFLS testing samples. Samples from affected individuals 52–55 (light grey) were used as WHIKERS discovery samples. Samples from affected individuals 56–59 (dark grey) were used as WHIKERS testing samples. Classification according to the ACMG guidelines

**Fig. 3:**
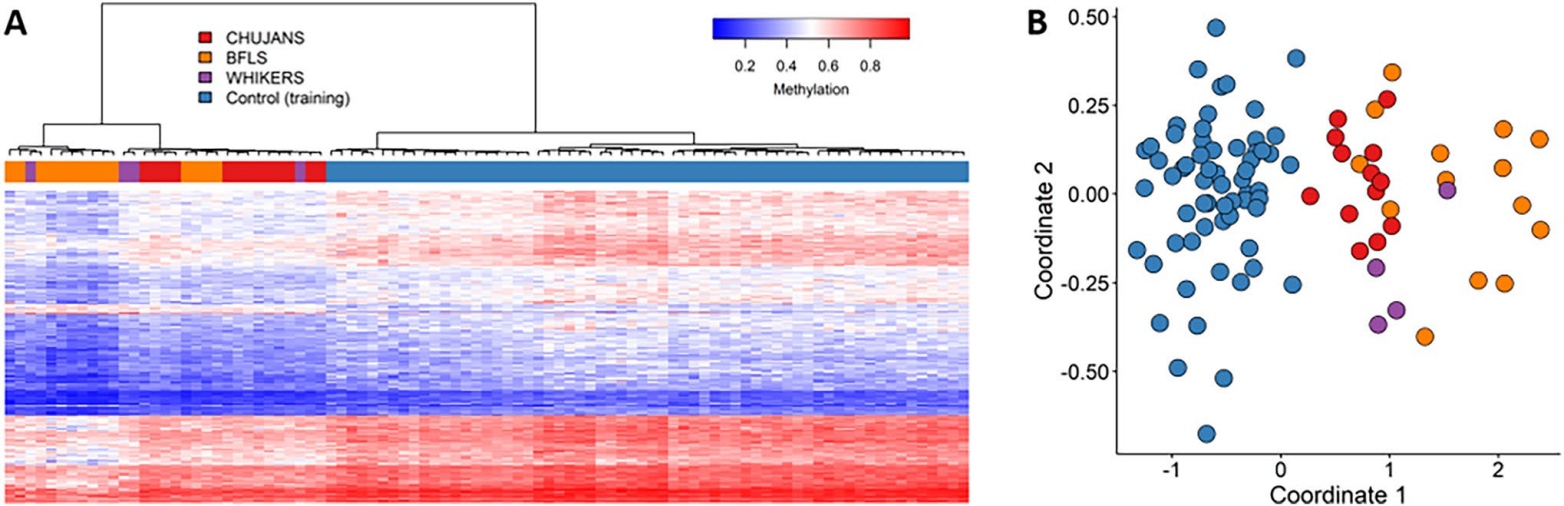
Evaluation of the episignature detected for the combined cohort using hierarchical clustering and MDS. **A** Hierarchical clustering was applied, where rows and columns represented the selected probes and samples, respectively. The color scale indicated methylation levels from blue (no methylation or 0) to red (full methylation or 1). On the heatmap pane, red, orange, purple, and blue colors represented CHUJANS, BFLS, WHIKERS, and control samples, respectively. This plot demonstrates that the affected individuals’ samples can be clearly distinguished from controls using the selected set of probes. **B** The MDS plot, depicted using the same color scheme as the heatmap for sample types, clearly showed separation of the combined cohort from the control group

Episignature analysis identified 213 differentially methylated CpG probes that successfully distinguished the CHUJANS, WHIKERS and BFLS discovery cohort samples from controls. Hierarchical clustering (heatmap) and MDS methods confirmed that the samples from the CHUJANS, WHIKERS and BFLS discovery cohorts clustered apart from controls based on differential methylation at the selected probes (Figs. 3A and 3B, respectively).

The robustness of the episignature was further assessed by plotting negative control samples (including unaffected individuals and individuals affected by other rare genetic disorders) alongside the case and control samples used for probe selection (Figure S4). The DNAm patterns of the CHUJANS, WHIKERS and BFLS testing cohort samples were visualized using the combined episignature, by applying hierarchical clustering and MDS. All the CHUJANS samples clustered together with the probe selection cohort samples, indicating the high sensitivity of the combined episignature. Two out of the four WHIKERS testing cohort samples matched the episignature. For the BFLS cohort, all of the testing samples, with the exception of two, did not match the combined episignature. We also created an SVM model using the 213 identified probes. The model was constructed by training the 31 samples (Tables 1 and 2) from the combined discovery cohort against the matched control cohort, 75% of other controls, and 75% of samples from other EpiSign™ V3 clinical classifier disorders within the EKD. The remaining 25% were allocated for testing. Subsequently, the constructed model was employed to test the remaining study samples (Figure S5, Tables 1 and 2). Individual 23, which was the only one in the CHUJANS testing set with a VUS in *PHIP*, clustered with the case samples that were used for probe selection. Therefore, the variant can be reclassified to LP according to the ACMG guidelines.

### Improving the classifier specificity

In an attempt to construct classifiers specific to CHUJANS, samples of 13 affected individuals with CHUJANS were used as the affected individual samples and 52 matched control individuals, samples of 14 affected individuals with BFLS, and samples of 4 affected individuals with WHIKERS were used as control samples (i.e., samples of BFLS and WHIKERS cohorts were added to the control set). As seen in Figure S6A, despite being used as control samples, both BFLS and WHIKERS samples, particularly the WHIKERS samples, still exhibit some degree of similarity in methylation pattern with the CHUJANS. Next, the samples of affected individuals with CHUJANS and WHIKERS were used as affected individuals’ samples and samples of matched controls and affected individuals with BFLS were used as control samples, but the samples of affected individuals with BFLS samples still clustered more closely to the samples of affected individuals with CHUJANS and WHIKERS (Figure S6B). The presence of a BFLS-specific methylation profile was also investigated by using the samples of individuals with BFLS as affected individuals’ samples and the samples of matched controls, affected individuals with CHUJANS, and affected individuals with WHIKERS as control samples, but no distinct separation was observed (Figure S65C).

### Functional comparison of CHUJANS relative to other episignature conditions

**Fig. 4:**
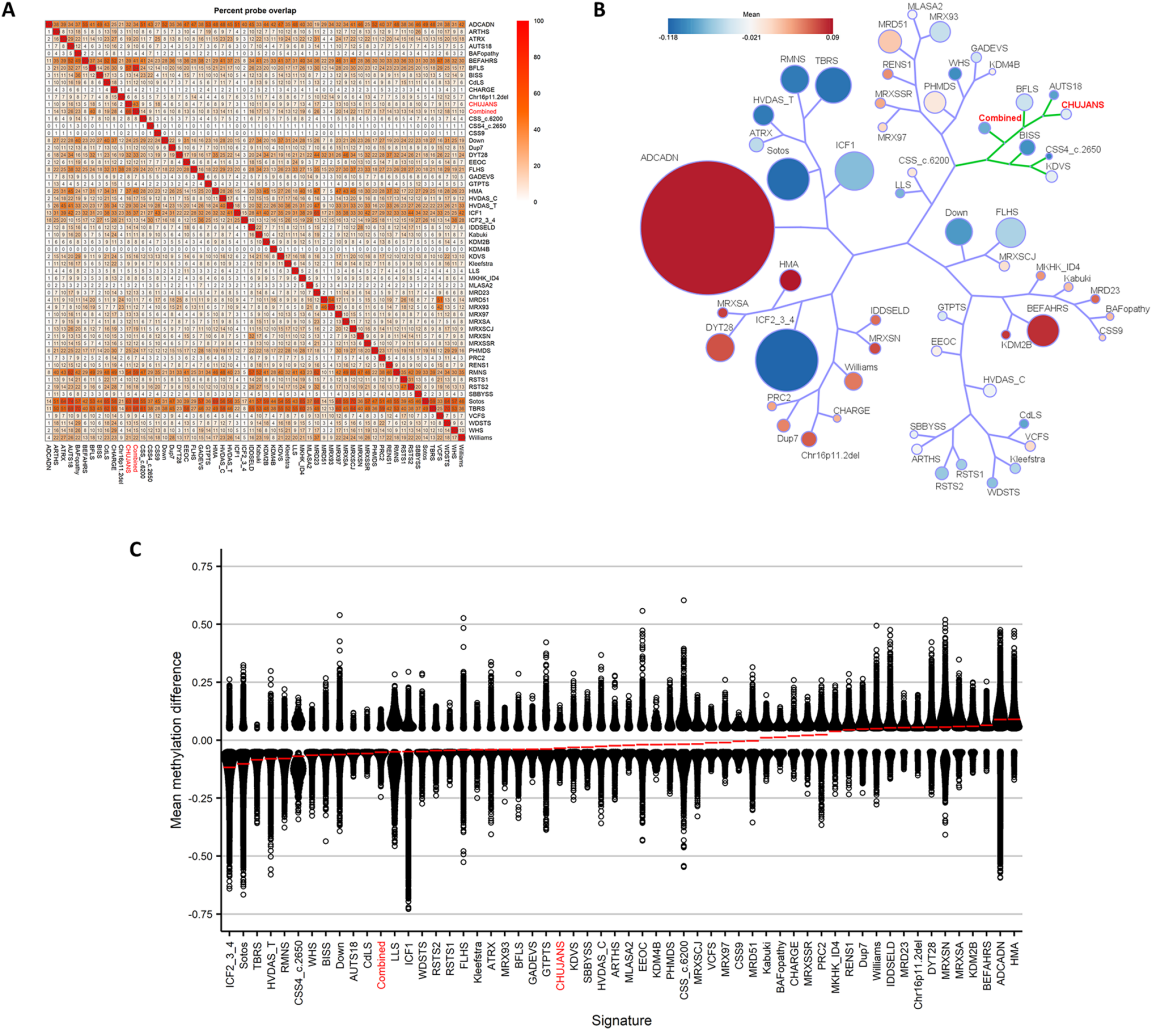
Assessment of the amount of DMPs shared between the CHUJANS and combined cohorts, and other syndromes with known episignatures. **A** Methylation probe overlap. The percentage of DMPs shared between disorders is shown on the color scale, ranging from white (0%) to red (100%). Each square in the graph represents the percentage of common probes between two syndromes, with the percentage of DMPs from the syndrome on the lower bar that also exist in the DMPs of the syndrome on the right-hand side bar. A high degree of overlap is observed between the CHUJANS, BFLS, and combined cohorts. **B** Relatedness assessment. A tree-and-leaf diagram is used, where each node represents a cohort, and syndromes with more similarity in methylation levels are located closer on the tree. Node size is related to the ratio of the number of DMPs to the total number of probes, while node color demonstrates the overall mean methylation difference in the corresponding cohort. The branch colored in green shows the one containing the study cohorts, indicating specifically the similarity in methylation levels of the CHUJANS, BFLS, and combined cohorts. **C** Comparison of the global mean methylation differences between syndromes with known episignatures. The plot demonstrates the overall hypomethylation of both CHUJANS and combined profiles, similar to that of BFLS. However, BFLS shows more hypomethylation compared to CHUJANS, and the combined cohort is more hypomethylated than both

With a criterion of a mean methylation difference > 5% and a corrected p-value of < 0.01, we identified 3843 DMPs for CHUJANS and 5916 DMPs for the combined cohort. We compared the percentage of DMP overlap of the CHUJANS and combined cohorts with those of EpiSign™ V3 clinical classifier disorders within EKD (Levy et al. 2022a) in Fig. 4A, and generated a tree-and-leaf diagram to compare the similarity of their methylation profiles in Fig. 4B. We quantified the mean global methylation levels of the CHUJANS and combined cohorts relative to other episignature cohorts. For each cohort, we calculated the average of mean methylation differences between the affected individuals and control groups across DMPs and demonstrated it with a red line (Fig. 4C). The CHUJANS and the combined CHUJANS-BFLS-WHIKERS episignature both show a predominantly hypomethylated profile.

Lastly, we performed annotation analyses of the identified DMPs for the CHUJANS and combined cohorts in the context of CpG islands (CGIs) and genes and compared this to the other cohorts in EKD. In comparison to the ‘default’ probes (> 50% DMPs inter_CGI), most of the CHUJANS and combined CHUJANS-BFLS-WHIKERS cohorts’ DMPs are present in CpG islands and shores (~ 75%) and in promoter/promoter + regions (Figure S7).

## Discussion

Genetic testing for NDDs, including genomic sequencing technologies, often results in the identification of novel rare variants with unknown impact on protein function, or VUS. Clinical interpretation relies on detailed clinical assessment, segregation analysis, functional and sometimes in silico studies to determine the pathogenicity of such variants. Although specific dysmorphic features are known for CHUJANS, and clinical assessment is therefore important, it is often challenging to resolve pathogenicity of *PHIP* variants. A better understanding of pathophysiology and additional functional and diagnostic technologies are therefore required. DNAm episignatures provide one such method enabling additional molecular diagnosis and reclassification of VUS in affected individuals with Mendelian disorders (Levy et al. 2022a; Aref-Eshghi et al. 2017; Verberne et al. xxxx).

We here demonstrate a novel episignature for affected individuals with CHUJANS. The CHUJANS episignature was discovered by analyzing a cohort of 13 affected individuals with likely pathogenic and pathogenic *PHIP* variants. Hierarchical clustering and MDS analyses of the training data reveal that samples from individuals with CHUJANS form a distinct cluster separate from controls, indicating the robustness of the episignature (Figs. 2 and 3).

In addition, as depicted in Figs. 4 and S6, it is evident that the CHUJANS cohort exhibits a noticeable degree of overlap in DNAm pattern with BFLS (Levy et al. 2022a). BFLS is a rare, X-linked, NDD, caused by pathogenic *PHF6* variants*.* Clinical features of BFLS are partially overlapping with CHUJANS and include hypotonia, DD, ID, behavioral problems, childhood onset obesity, visual anomalies, and dysmorphic features such as large ears with fleshy earlobes (Crawford et al. 2006; Turner et al. 2004). An interaction between PHIP and PHF6 has previously been described (Morgan et al. 2017). Additionally, we noticed that four samples of affected individuals with WHIKERS, caused by variants in *DDB1,* had a DNAm pattern similar to the CHUJANS episignature. This might be explained by the fact that PHIP is known to form a protein complex with DDB1 and CUL4 (Townsend et al. 2021; Lee and Zhou 2007) and clinical overlap exists between *DDB1*-, *CUL4*- and *PHIP*-associated disorders (White et al. 2021). Clinical features of WHIKERS include hypotonia, DD, ID, obesity and dysmorphic features such as synophrys, deep-set eyes, fleshy forward-facing earlobes, and a short nose (White et al. 2021).

Due to the observed high degree of overlap in clinical features and the DNAm patterns of CHUJANS, BFLS and WHIKERS samples as well as the interaction of the corresponding genes (Aref-Eshghi et al. 2018a), we further investigated and detected a highly sensitive and specific combined episignature for CHUJANS, BFLS and WHIKERS. Subsequently, a combined classifier was developed that exhibited a higher sensitivity in detecting affected individuals with CHUJANS (Figure S5). Within the CHUJANS cohort, individual 27 was particularly interesting. This affected individual remained unresolved in the EKD, without a known variant, yet received a high MVP score on both the CHUJANS and the combined classifiers. This was verified through hierarchical clustering and MDS. Consequently, further investigation of this individual’s DNA revealed a likely pathogenic *PHIP* variant, emphasizing the robustness and enhanced utility of episignatures in identifying molecular markers, and thus, confirming clinical diagnoses. The extent of overlap in DMPs between the CHUJANS cohort, the combined CHUJANS-BFLS-WHIKERS cohort and other known episignatures was investigated. The CHUJANS, BFLS and the combined cohort demonstrated a high degree of overlap in their DNAm patterns. It is worth noting that samples of affected individuals from our CHUJANS and WHIKERS cohorts demonstrated a higher degree of similarity in their DNAm patterns compared to the samples of our affected individuals with BFLS (Figures S2 and S6). The presence of isolated, CHUJANS-specific and BFLS-specific episignatures was investigated, but not (yet) found (Figure S6).

Other combined episignatures, such as for the BAFopathy (genes: *ARID1A, ARID1B, SMARCB1, SMARCA2* and *SMARCA4*) episignature, are already proving their clinical utility (Aref-Eshghi et al. 2018b). For future individuals with a positive diagnostic CHUJANS-BFLS-WHIKERS episignature, we thus recommend analysis of the *PHIP*, *PHF6* and *DDB1* genes in order to discover the causative genetic defect. Not all affected individuals in the BFLS and WHIKERS testing cohorts in our study had a positive CHUJANS-BFLS-WHIKERS episignature. None of the females with *PHF6* variants had a positive episignature, which might have to do with the fact that BFLS is an X-linked disorder and sex-specific phenotypical differences have been described for affected individuals with *PHF6* variants (Gerber et al. 2022). Whether a female-specific BFLS episignature can be identified, remains to be elucidated.

Additionally, none of the four included individuals with *PHF6* VUS matched the combined episignature, and two out of three included individuals with a *DDB1* VUS did not match it either. These individuals are currently left without definitive diagnosis and we hope that follow-up studies can provide more clarity about the pathogenicity of these variants.

Apart from confirmation of the diagnosis and understanding the underlying pathology, these episignatures can also lead to disorder-specific clinical recommendations. Importantly, due to the involvement of PHIP in the leptin-melanocortin pathway, affected individuals with CHUJANS who suffer from therapy resistant obesity can participate in a clinical trial with an anti-obesity drug (setmelanotide (MC4R-agonist), ClinicalTrials.gov identifier: NCT04963231). The clinical and molecular overlap and combined episignature for CHUJANS, BFLS and WHIKERS, could indicate an involvement of PHF6 and DDB1 in the leptin-melanocortin pathway (Yeo et al. 2021). This needs to be further elucidated, so that affected individuals with BFLS and WHIKERS suffering from obesity could potentially benefit from these personalized medicine options in the future.

In conclusion, we here present a distinct episignature for Chung-Jansen syndrome, caused by pathogenic *PHIP* variants. In accordance with the clinical and molecular similarities between CHUJANS, BFLS, and WHIKERS, we provided and confirmed an overlapping sensitive and specific episignature for these three syndromes. Furthermore, we showcased the efficacy of episignatures in confirming the pathogenicity of VUS within our cohort, thereby assisting in the provision of a conclusive molecular diagnosis based on the episignature. This process contributes significantly to establishing diagnoses for affected individuals with CHUJANS, WHIKERS or BFLS and enhances our comprehension of its underlying pathophysiology.

### Supplementary Information

Below is the link to the electronic supplementary material.

Supplementary file1 (DOCX 17 KB)

Supplementary file2 (PDF 2328 KB)

Supplementary file3 (XLSX 121 KB)

## Data Availability

All data generated or analyzed during this study are available from the corresponding author on reasonable request.
