# Supplementary material for: The detection of a strong episignature for Chung–Jansen syndrome, partially overlapping with Börjeson–Forssman–Lehmann and White–Kernohan syndromes
Source: Hum Genet. 2024 May 24;143(6):761–73. doi: 10.1007/s00439-024-02679-w (PMC11186873; doi:10.1007/s00439-024-02679-w)
Supplement: Supplementary file 2 — Supplementary file2 (PDF 2328 KB) [file 439_2024_2679_MOESM2_ESM.pdf]

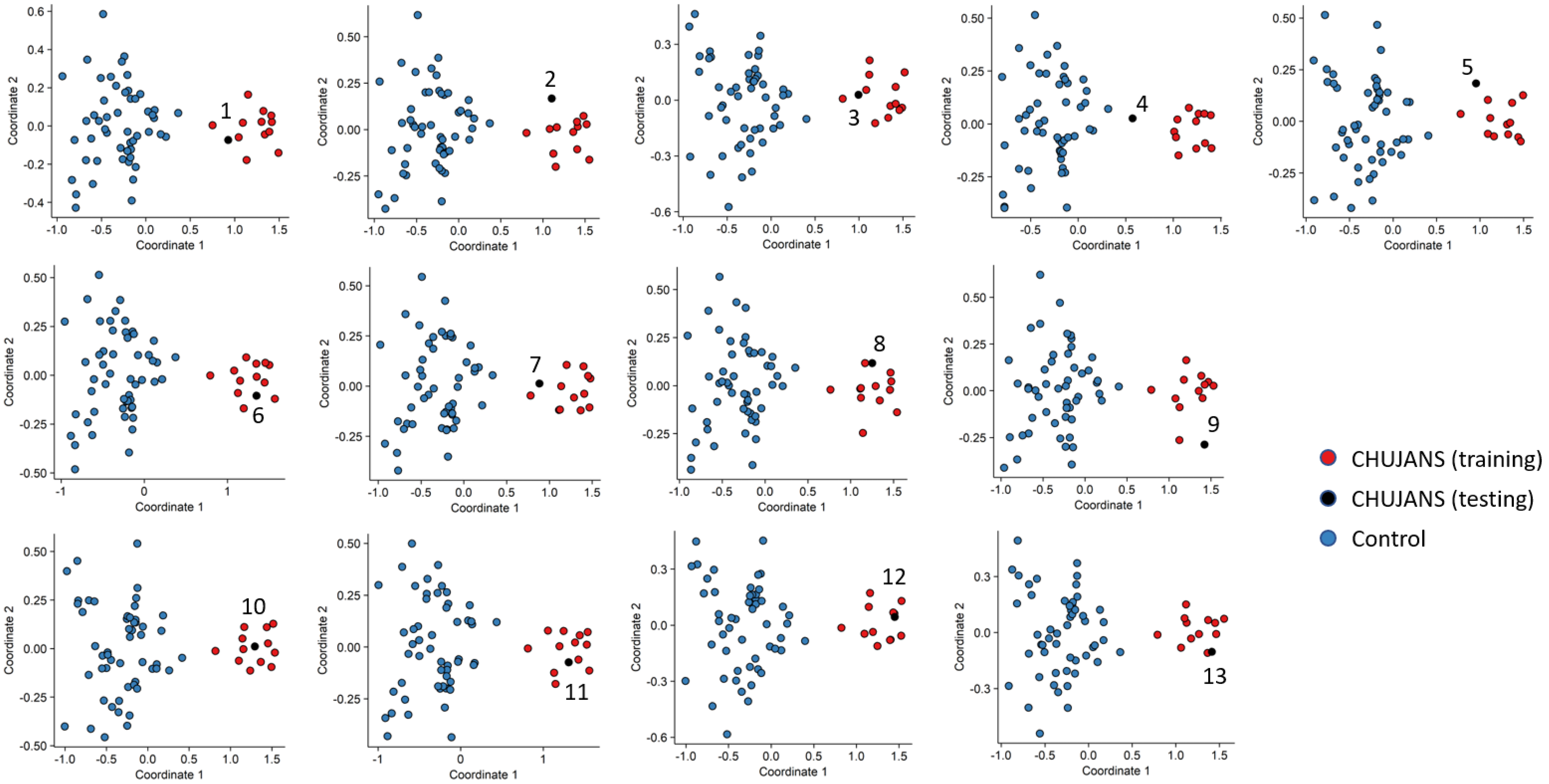

Figure S1. LOOCV for the CHUJANS cohort. In all rounds of LOOCV, the CHUJANS testing sample (represented by a black circle) clustered with the CHUJANS samples used for probe selection (represented by red circles) and separate from the control individuals (represented by blue circles), confirming the reproducibility of the episignature.

A

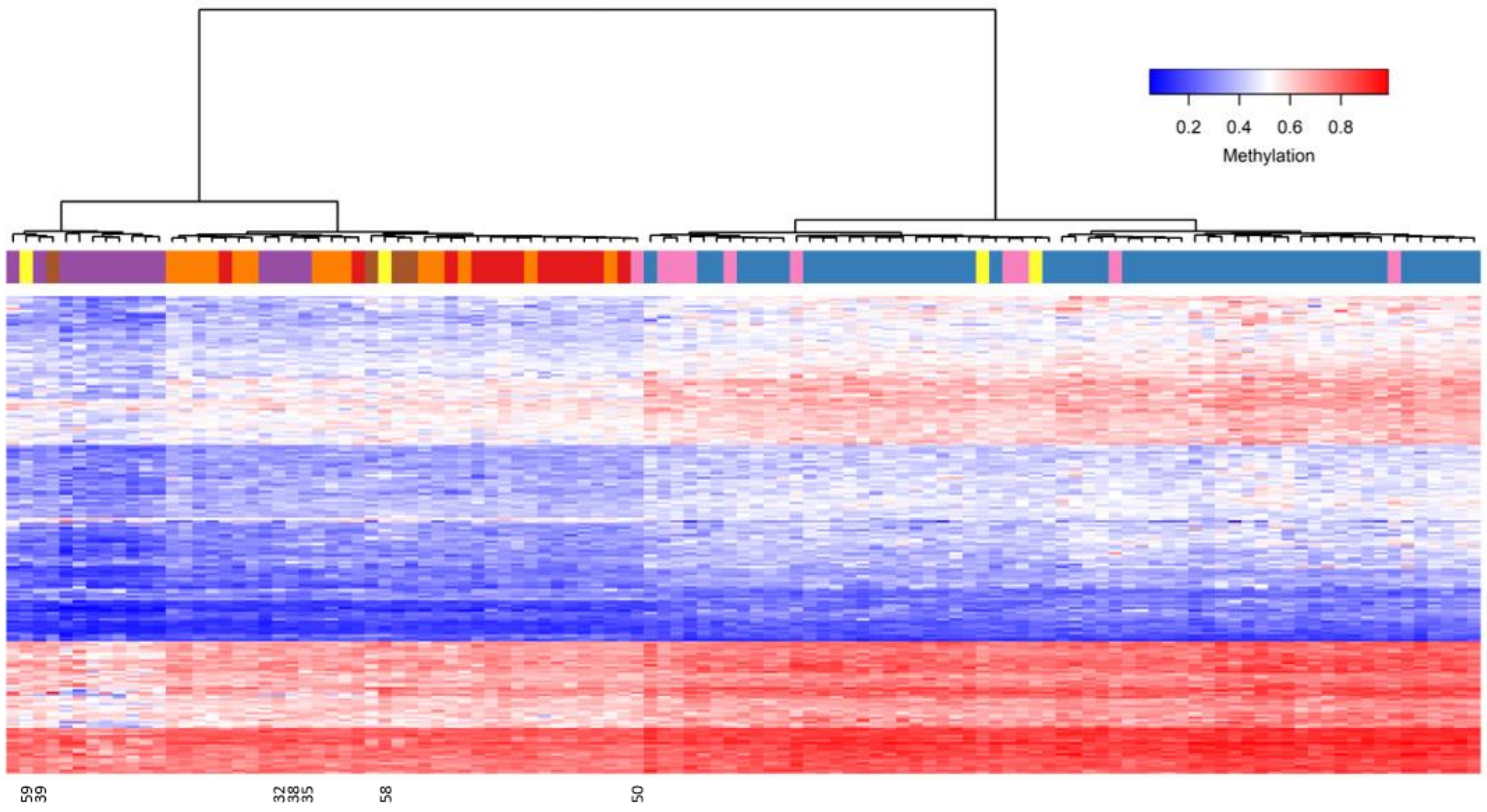

B

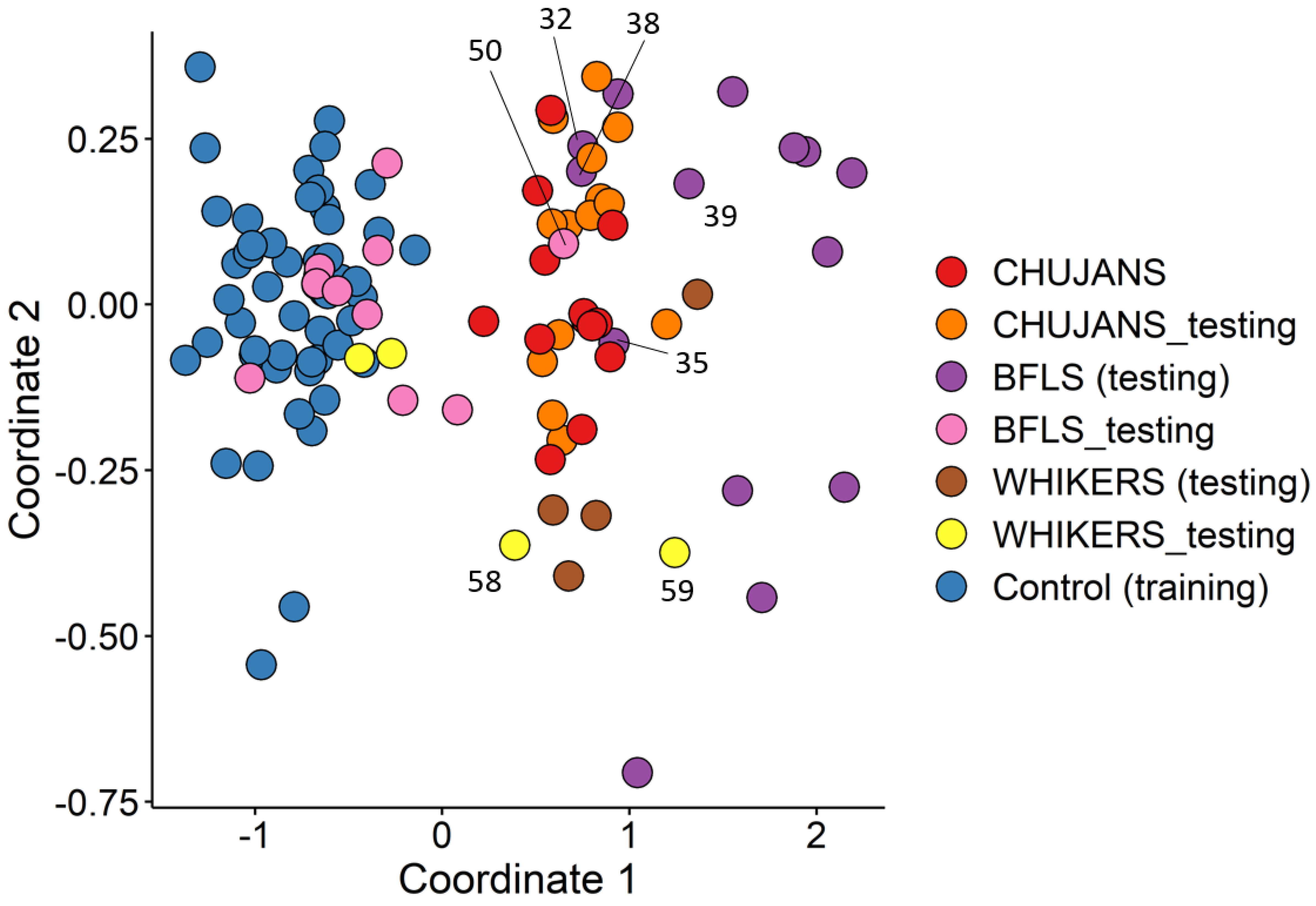

Figure S2. A The addition of all study samples to the hierarchical clustering plot for the CHUJANS episignature. The heatmap was generated using the 212 selected probes, and additional study samples were plotted alongside the CHUJANS and control individuals used for probe selection. The CHUJANS and control samples used for probe selection were indicated with red and blue, respectively, and the CHUJANS testing, BFLS, BFLS testing, WHIKERS, and WHIKERS testing samples were indicated with orange, purple, pink, brown, and yellow, respectively. All the CHUJANS testing samples clustered with the CHUJANS group used for probe selection. The WHIKERS samples, two of the WHIKERS testing samples, four of the BFLS samples, and one of the BFLS testing samples showed variable degrees of similarity with the CHUJANS methylation pattern. **B** The addition of all study samples to the MDS plot. The sample types were indicated using the same colors as shown in Figure S2A. Every sample from the CHUJANS testing group formed clusters with the CHUJANS samples used for probe selection. Among the remaining samples, all the WHIKERS samples, two from the WHIKERS testing group, four from BFLS, and one from the BFLS testing group exhibited varying levels of resemblance to the CHUJANS methylation pattern.

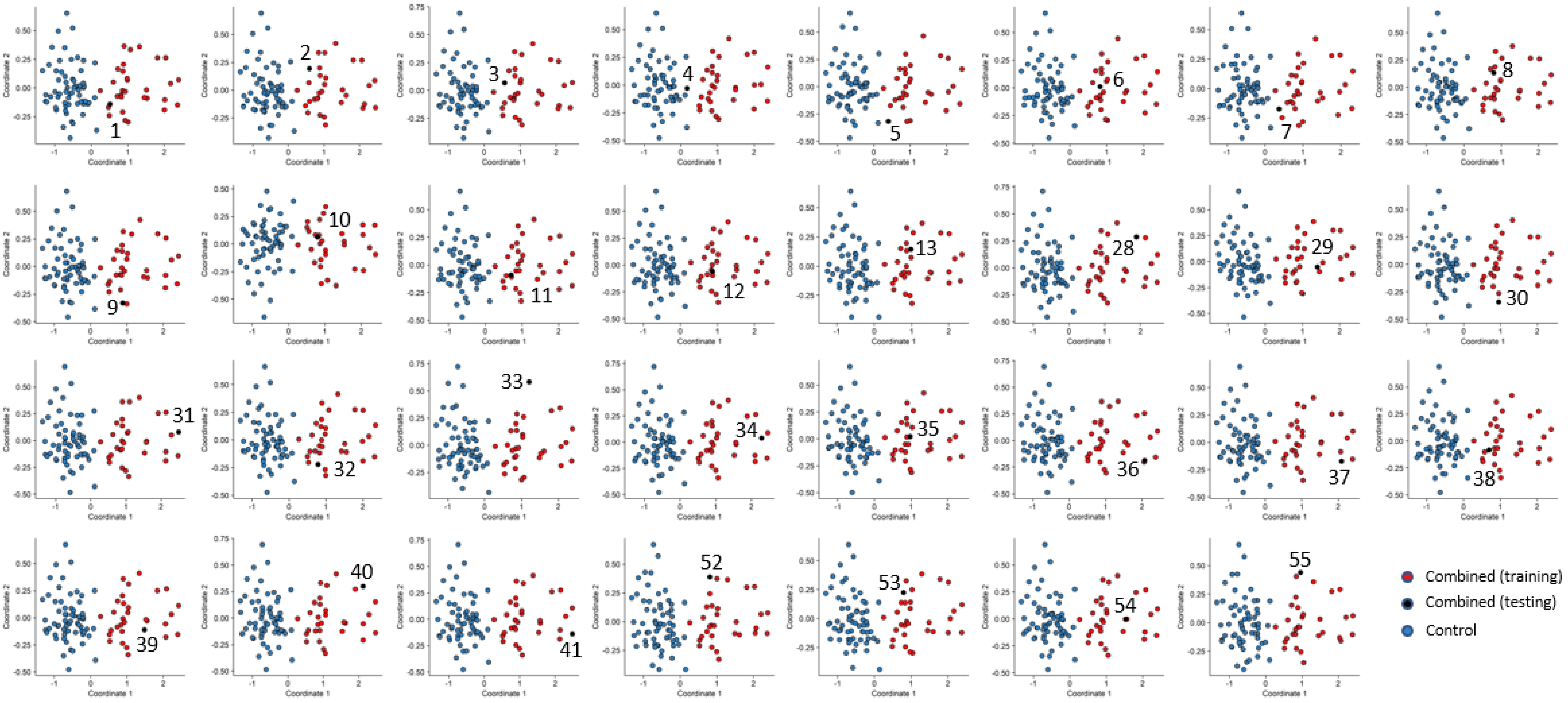

Figure S3. LOOCV for the combined CHUJANS-WHIKERS-BFLS cohort. In all iterations of LOOCV, the testing sample (black circle) clustered with the CHUJANS samples used for probe selection (represented by red circles) and separated from the control individuals (blue circles), demonstrating the reproducibility of the episignature.

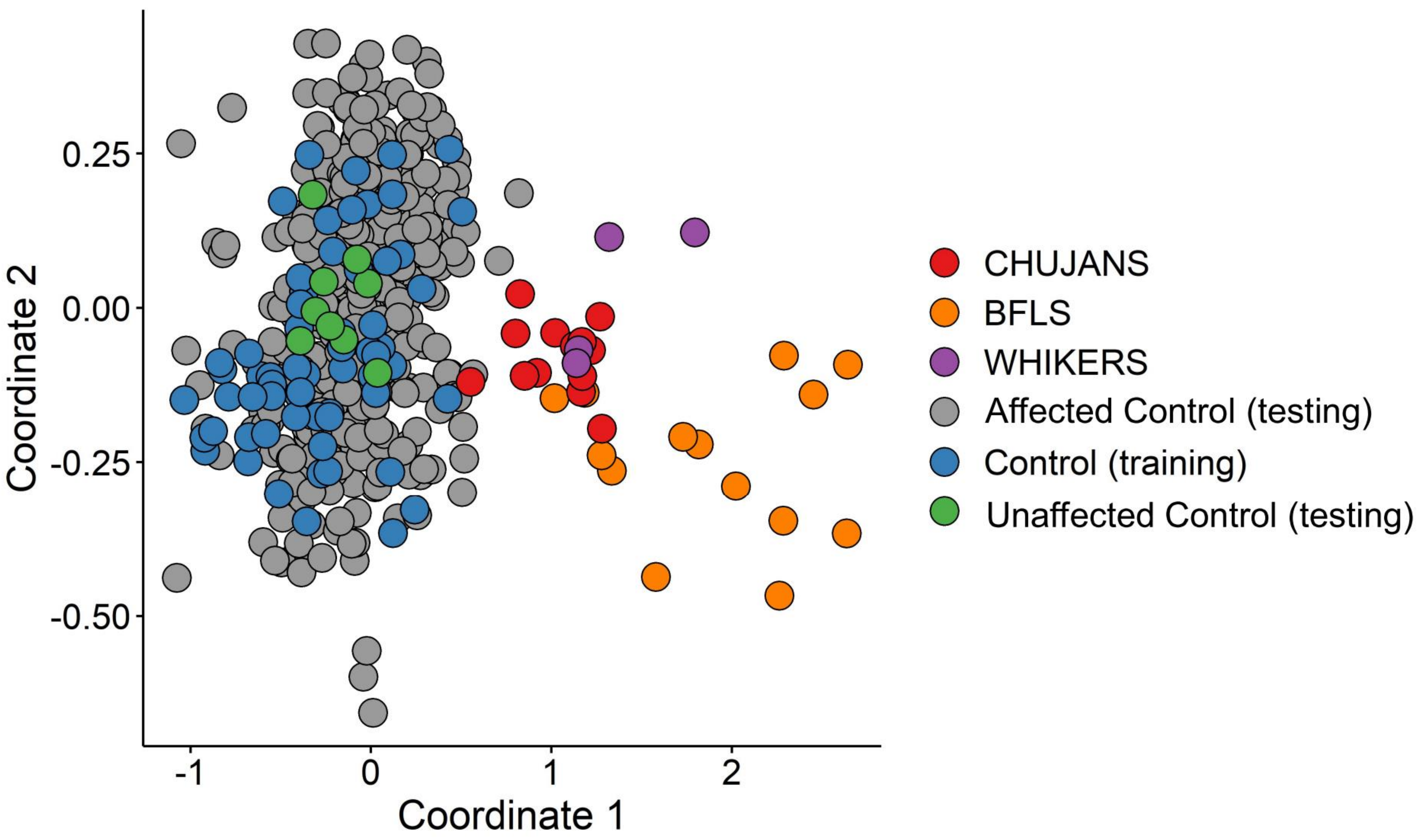

Figure S4. Negative controls were included to further test the robustness of the combined episignature. Samples from other disorders (displayed with grey circles) and unaffected control samples (indicated with green circles) were plotted alongside samples used for probe selection (CHUJANS, BFLS, WHIKERS, and matched control samples, indicated with red, orange, purple, and blue circles, respectively). The testing samples clustered with the probe selection control samples, providing evidence for the specificity of the selected probes to the combined cohort.

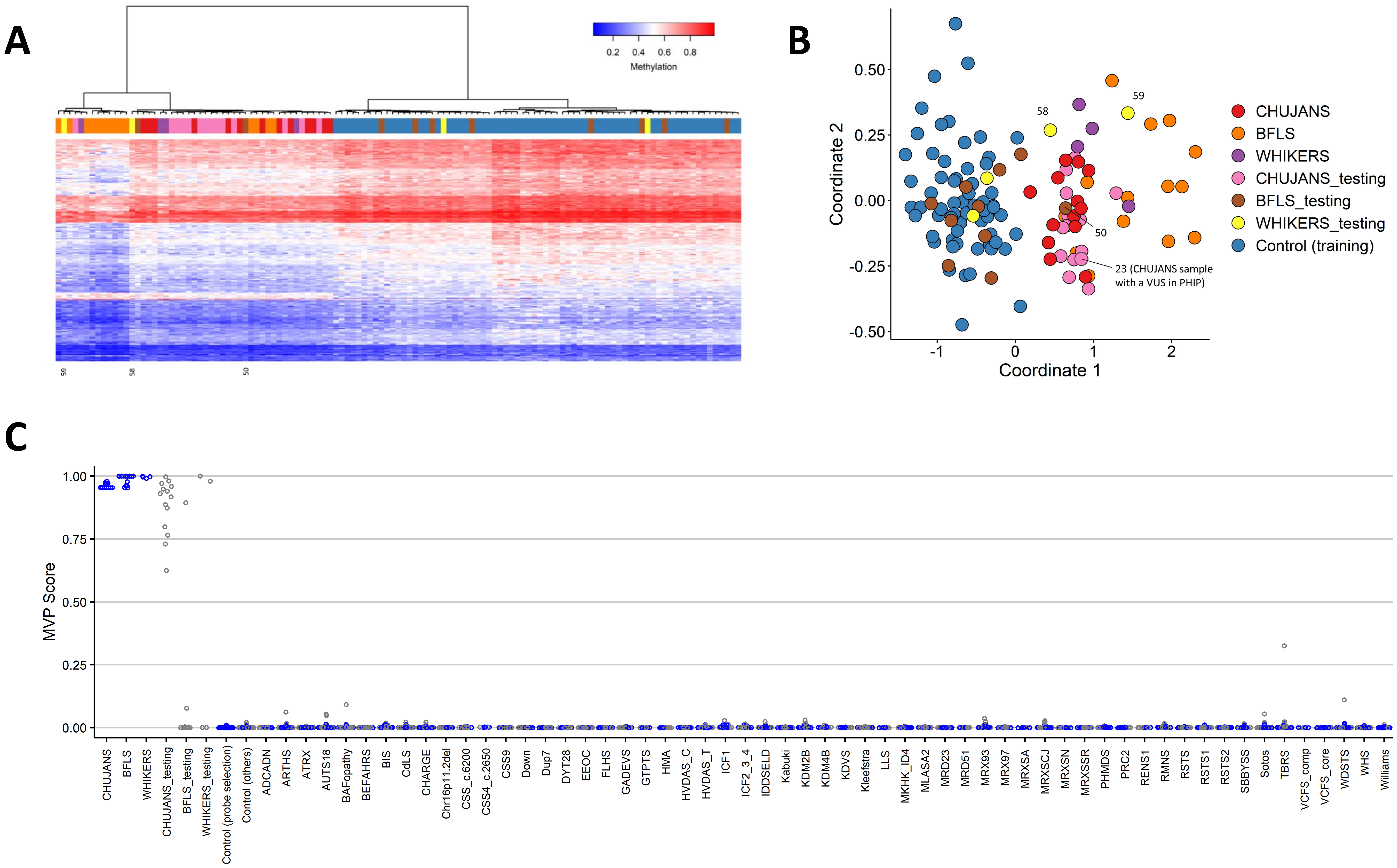

Figure S5. A. B The addition of all study samples to the hierarchical clustering plot. The heatmap was updated with the remaining study samples. The plot included samples used for probe selection (i.e., CHUJANS, BFLS, WHIKERS, and control individuals represented by red, orange, purple, and blue, respectively) and additional study samples (i.e., CHUJANS testing, BFLS testing, and WHIKERS testing samples represented by pink, brown, and yellow, respectively). It was observed that all CHUJANS testing samples, two WHIKERS testing samples, and one BFLS testing sample clustered with samples from the combined cohort used for probe selection. The CHUJANS and WHIKERS cohorts demonstrated a higher degree of similarity in methylation patterns compared to the BFLS set. C SVM model performance for the combined CHUJANS-WHIKERS-BFLS cohort. The MVP scores, generated by the SVM classifier, represent the training and testing samples as blue and grey circles, respectively. The low scores of testing samples from other disorders demonstrate the model's high specificity. All CHUJANS testing samples, two BFLS testing samples and two WHIKERS testing samples received MVP scores greater than 0.5. This indicates the similarity of their methylation pattern to that of the combined cohort.

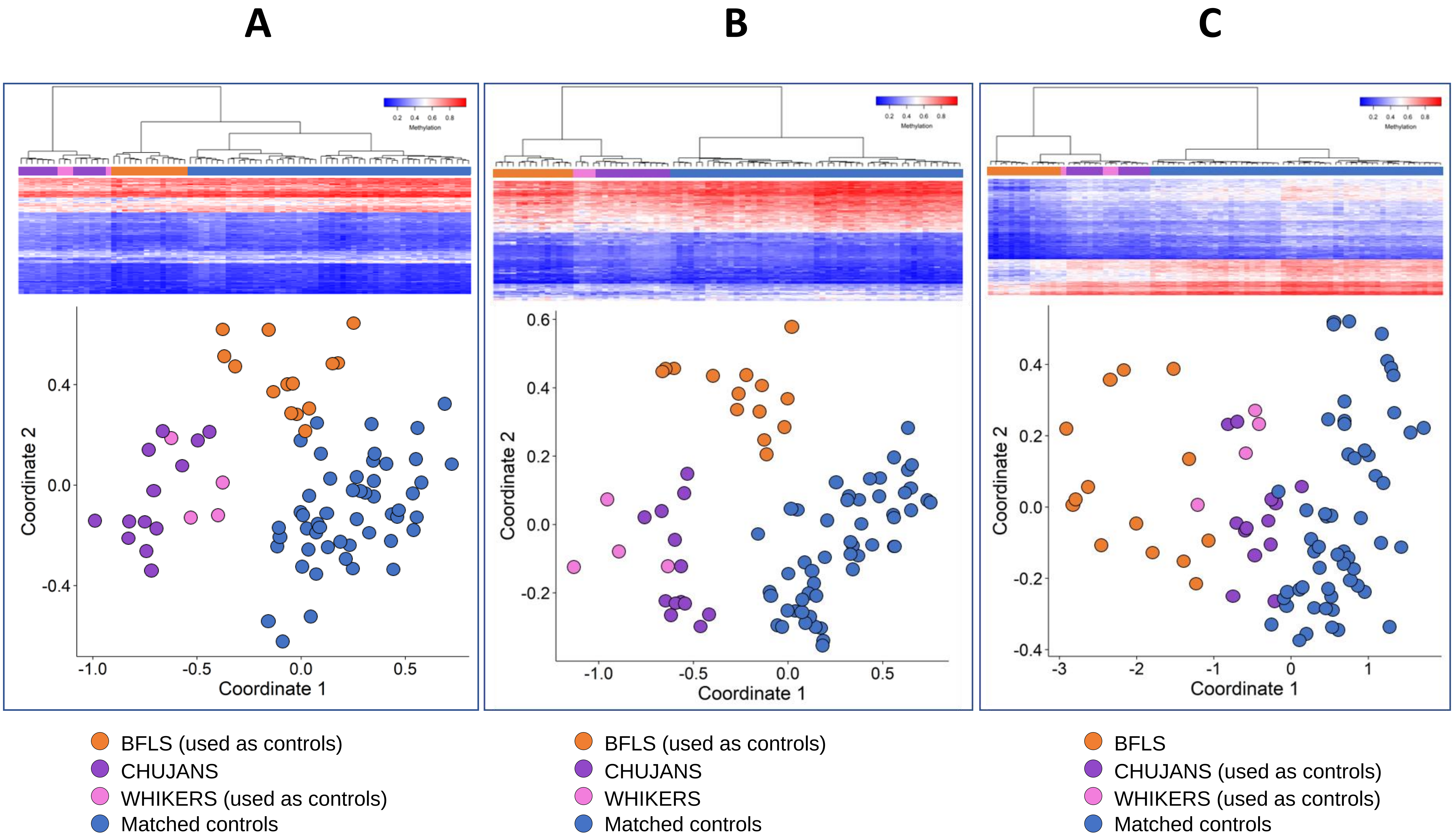

Figure S6. A Investigating the presence of a CHUJANS-specific episignature. CHUJANS, BFLS, WHIKERS, and control samples are represented with purple, orange, pink, and blue circles, respectively. Probe selection was performed using CHUJANS samples as case samples and BFLS, WHIKERS, and matched control individuals as control samples. No CHUJANS-specific episignature was detected, and, in particular, WHIKERS samples demonstrated a high degree of overlap with the CHUJANS' methylation pattern. **B.** Investigating the presence of a CHUJANS/WHIKERS-specific episignature. The sample categories were highlighted using the identical color scheme as demonstrated in Figure S6A. Probe selection was conducted using CHUJANS and WHIKERS samples as case samples and BFLS and matched control samples as control individuals. BFLS samples clustered more closely with the CHUJANS-WHIKERS cluster, indicating some degree of similarity in methylation pattern. **C.** Investigating the presence of a BFLS-specific episignature. The same color scheme as presented in Figure S6A was utilized to indicate the sample categories. Probe selection was performed by using BFLS samples as cases and CHUJANS, WHIKLERS, and matched controls as the control group. The CHUJANS and WHIKERS samples clustered together between the BFLS and control groups, and no BFLS-specific episignature was detected.

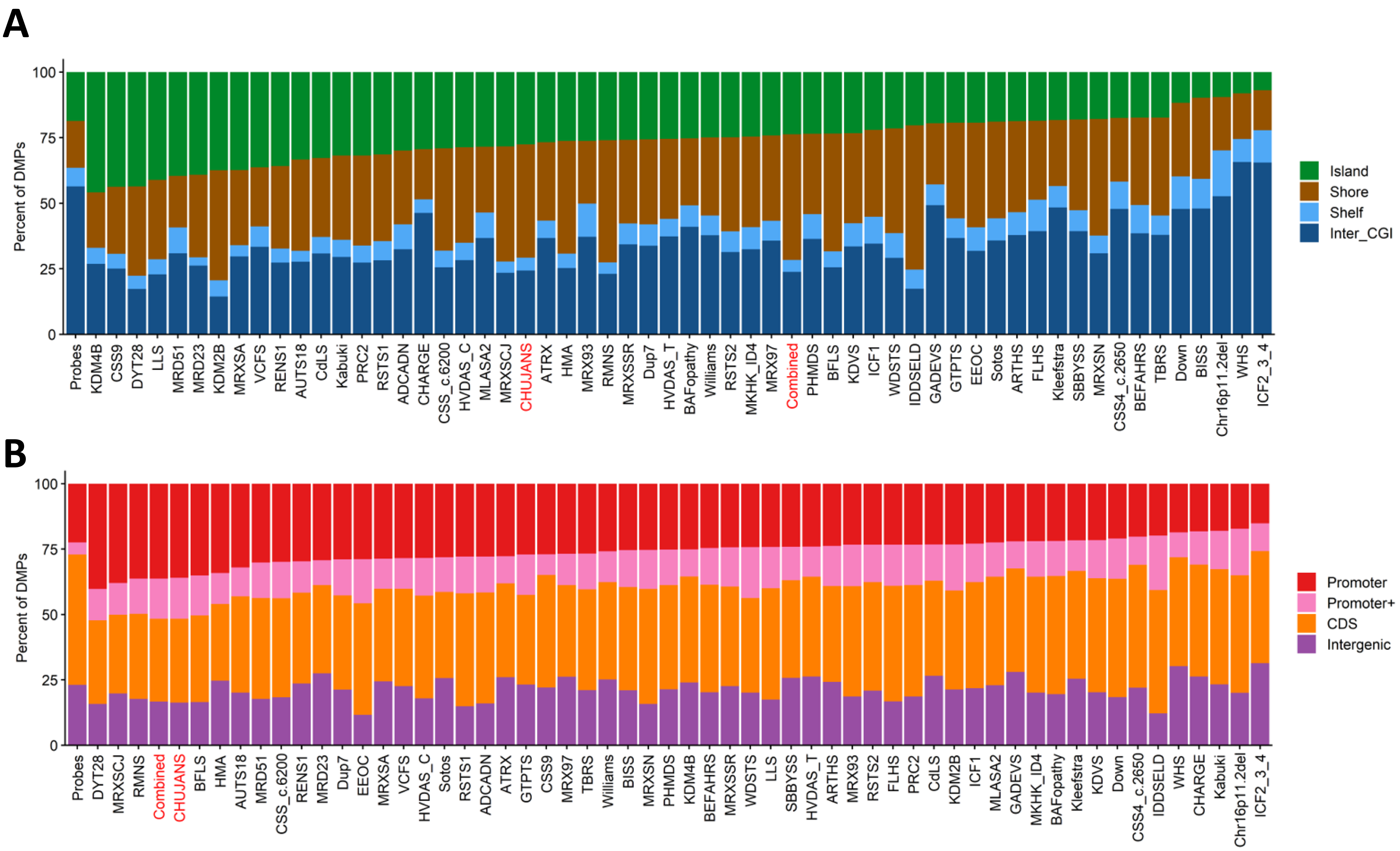

Figure S7. A Annotation of DMPs in relation with CpG islands for the CHUJANS and combined cohorts. The "probes" column illustrates the "default" distribution of all probes included in both 450k and EPIC arrays, which remain after the initial probe filtering process. Annotation includes different regions: CpG islands, shores (within 2kb of a CpG island boundary), shelves (within 2-4kb of a CpG island boundary), and inter_CGI (other genomic regions). Approximately 75% of DMPs in both the CHUJANS and combined cohorts were located on a CpG island or a shore, while the "default" ratio is around 40% for all probes. **B** Annotation of DMPs in relation with genes for the CHUJANS and combined cohorts. Annotation includes different regions: promoter (within 1 kb upstream of the transcription start site) and promoter+ (1-5kb upstream of the transcription start site), as well as the coding sequence (CDS). In both the CHUJANS and combined cohorts, approximately 50% of DMPs were annotated to be in a promoter or promoter+ region, whereas for all probes, the "default" number was around 25%.
